# Supplementary material for: Synthesis of Manganese Zinc Ferrite Nanoparticles in Medical-Grade Silicone for MRI Applications
Source: Int J Mol Sci. 2023 Mar 16;24(6):5685. doi: 10.3390/ijms24065685 (PMC10059734; doi:10.3390/ijms24065685)
Supplement: Supplementary file 1 [file ijms-24-05685-s001.zip › ijms-2223658-supplementary.pdf]

International Journal of
Molecular Sciences

MDPI

Supplementary Materials

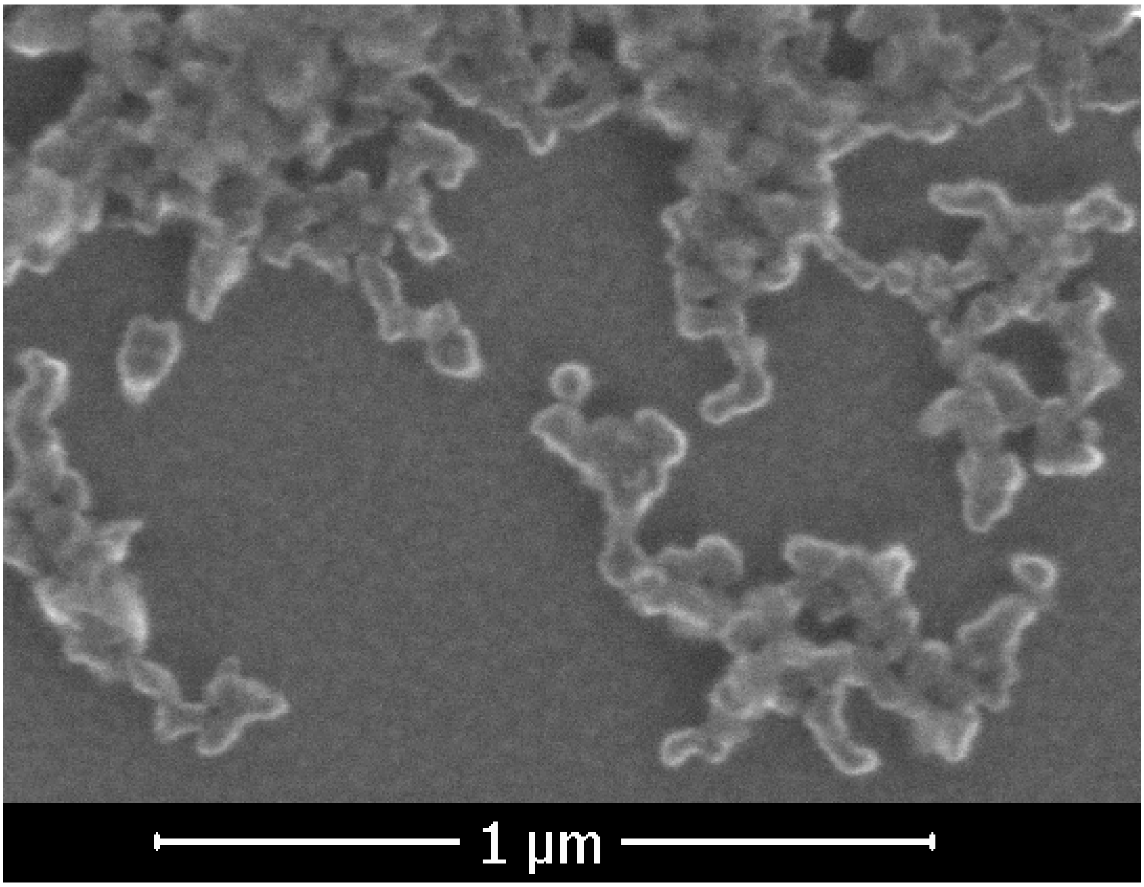

Figure S1. A representative SEM image, showing the morphology of nanoparticles grown in silicone. Scale bars are 1.0 µm.

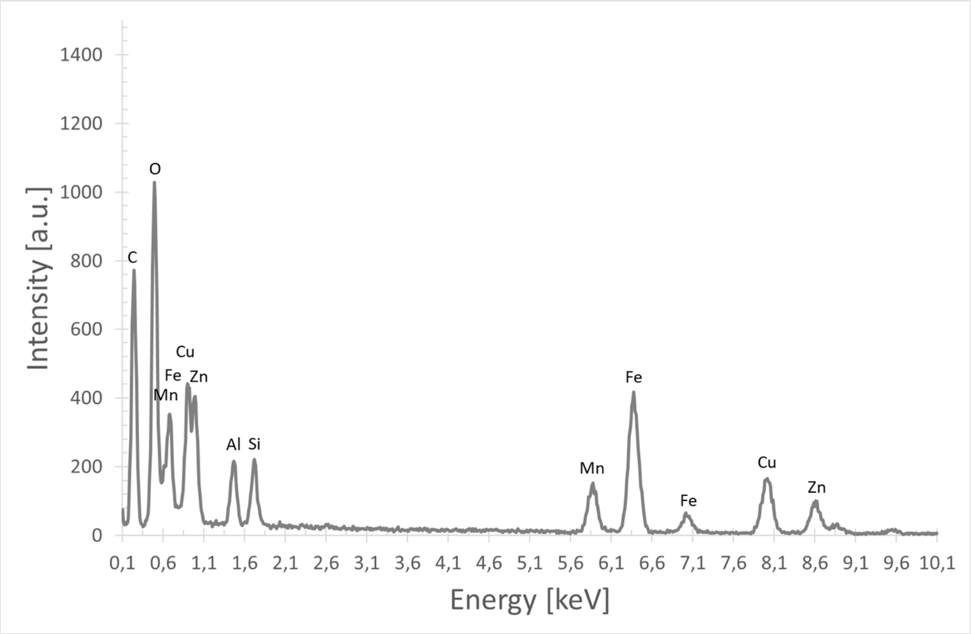

Figure S2. Energy-dispersive X-ray spectroscopy (EDS) of the MnZn ferrite revealing the chemical composition of the material.

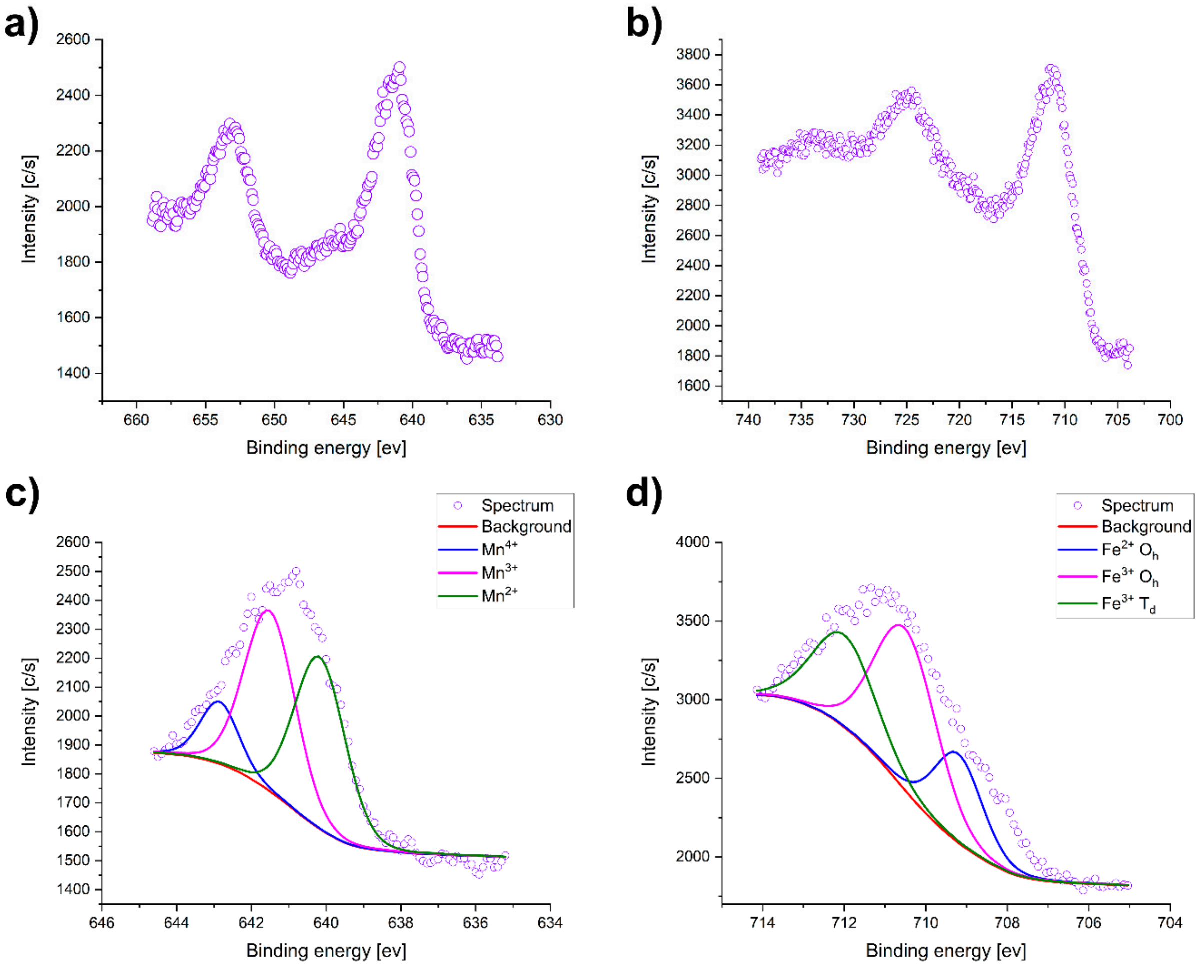

Figure S3. XPS spectra of the obtained nanoparticles in silicone: **(a)** The Mn 2p spectrum. **(b)** The Fe 2p spectrum. **(c)** The deconvoluted high-resolution Mn $2p_{3/2}$ XPS spectra from MnZn ferrite in silicone. **(d)** The deconvoluted high-resolution Fe $2p_{3/2}$ XPS spectra.
